# Bridging evidence, policy, and practice to strengthen health systems for improved maternal and newborn health in Pakistan

**DOI:** 10.1186/s12961-015-0034-7

**Published:** 2015-11-25

**Authors:** Atsumi Hirose, Sarah Hall, Zahid Memon, Julia Hussein

**Affiliations:** 1 Immpact, University of Aberdeen, Foresterhill, Health Sciences Building, Aberdeen, AB25 2ZD UK; 2 Research and Advocacy Fund, Islamabad, Pakistan; 3 Greenstar Social Marketing, Karachi, Pakistan; 4 Immpact, University of Aberdeen, Aberdeen, UK

**Keywords:** Advocacy, Evidence-informed policy, Health systems, Implementation research, Maternal health, Newborn health, Pakistan, Policy uptake

## Abstract

Policy and decision making should be based on evidence, but translating evidence into policy and practice is often sporadic and slow. It is recognised that the relationship between research and policy uptake is complex and that dissemination of research findings is necessary, but insufficient, for policy uptake. Political, social, and economic context, use of (credible) data and dialogues between and across networks of researchers and policymakers play important roles in evidence uptake. Advocacy is the process of mobilising political and public opinions to achieve specific aims and its role is crucial in mobilising key actors to push for policy uptake. Advocacy and research groups (i.e. those who would like to see research evidence used by policymakers) may use different approaches and tools to stimulate the diffusion of research findings. The use of mass- and social media, communication with study participants, and the involvement of stakeholders at the early stages of research development are examples of the approaches that can be employed to stimulate diffusion of evidence and increase evidence uptake. The Research and Advocacy Fund (RAF) for Maternal and Newborn Health (MNH) worked within the health system context in Pakistan with the aim of espousing the principles of evidence, advocacy, and dissemination to improve MNH outcomes. The articles included in this special issue are outputs of RAF and highlight where RAF’s approaches contributed to MNH policy reforms. The papers discuss critical health system issues facing Pakistan, including service delivery components, demand creation, equitable access, transportation interventions for improved referrals, availability of medicines and equipment, and health workforce needs. In addition to these tangible elements, the health system ‘software’, i.e. the power and the political and social contexts, is also represented in the collection. These articles highlight three considerations for the future: the growing importance of implementation research, the crucial need for participation and ownership, and the recognition that policymaking can be ‘informed’ by rather than ‘based-on’ evidence. The future challenge will be to continue the momentum RAF has created and to welcome a new era of health, wealth, and growth for Pakistan.

## Research, dissemination, and advocacy for policy uptake

Policy- and decision-making should be based on evidence [1], yet, translating evidence into policy and practice is often sporadic and slow [2]. In the United Kingdom, the need for policymakers to increase their reliability on evidence was debated and thus evidence-based policymaking became popularised during the late 1990s, which coincided with calls for greater investment into research in health in resource-poor countries [3, 4]. This lead to the creation of the Global Forum for Health Research, aiming to expand the use of evidence in policy, particularly for the benefit of resource-limited countries. There has been growing understanding of how to reduce the gap between what we know (evidence) and what is being done (practice) in international health and development. The slow progress towards the Millennium Development Goals (MDGs) has resulted in urgent calls to close the gap as demonstrated in three key international meetings: the Mexico Statement on Health Research in 2004, the World Health Assembly resolution in 2005, and the Bamako Call to Action on Research for Health in 2008. These have all recognised the role of research in closing the knowledge–practice gap and improving health outcomes. Consequently, knowledge translation and implementation research have become buzzing fields of research over the last decade.

The relationship between research and policy uptake is complex; few people would now defend the simplistic model of linear causality between the two and realisation of its complexity is growing. According to Grimshaw et al. [5], translation of knowledge into policy ensures that “*stakeholders are aware of and use research evidence to inform their health and healthcare decision-making*”. Contributions from social or political science fields suggest that three factors play important roles in influencing policy uptake: (1) political, social, and economic contexts; (2) utilisation of (credible) data; and (3) researcher and policymaker networks [6]. A multiplicity of actors is therefore involved in evidence uptake: policymakers, programme implementers, healthcare providers, patients, family members and other informal carers, researchers, and the medical and pharmaceutical industries.

Advocacy is the process of mobilising political and public opinions to achieve specific aims. Its role is crucial in mobilising key actors to push for evidence uptake and greater accountability. Advocacy groups or organisations include wide ranging groups of people with differing motives and purposes. For example, patient support groups may be instrumental in highlighting the gaps in treatment, care, and support to patients [7] which require changes, raising public awareness of specific diseases (for early detection and treatment), combating stigma and prejudice associated with certain diseases, or advocating for human rights [8]. Professional associations may provide their expert opinions to influence or ‘educate’ policymakers and patients [9]. In international health and development, international organisations, civil society organisations, non-governmental organisations (NGOs), and even national governments have influenced both public awareness and policymaking. In global health in particular, effective advocacy has been a determining factor in bringing what were once invisible health issues into global policy agendas [10-12].

Dissemination and diffusion of research findings is necessary for the transfer of evidence to practice and to influence policymaking [13]. Yet, dissemination by itself is not sufficient for policy uptake. Different approaches and tools need to be employed to stimulate the diffusion of research findings and engage a broader group of constituents so that they cross the boundary between science and politics or between scientists and lay people. The approaches used by actors who generate evidence (researchers or research institutions) include engaging with mass- [14] and social media and communicating with study participants [15], which is important as it empowers local communities to make decisions about their own health and to act upon them. Capacity-building of those who would like to see research evidence used by policymakers (advocacy organisations) is also warranted to ensure that research findings are shared, with fair credibility and without distortion and exaggeration, for ‘healthy’ knowledge translation, and to hold decision makers accountable.

## The health system: an essential backdrop for improving maternal and newborn health (MNH) outcomes

Once called a neglected tragedy as epitomised by Rosenfield and Maine’s seminal article [16], maternal mortality has now gained importance in the international health agenda, most notably in the form of the maternal mortality ratio included as a highly visible indicator to measure progress towards MDG-5. Closely related to maternal well-being is the health of the newborn and child, the improvement of which is addressed by MDG-4. There is wide agreement that ‘we know what works’ to avert maternal mortality and disability [17], but how to deliver effective interventions to women who need them is a key topic still debated within the global safe motherhood community. In order to achieve this, a functioning health system that is able to provide emergency obstetric care with a well-functioning referral system has been called for [18, 19]. Functioning health systems capable of improving health outcomes and strengthening maternity care for mothers and their children can only be achieved through adequate financing (to form a health system) as well as through the linking of health, referral, and community services in order to provide quality obstetric care within the health system [19]. The six building blocks of a health system, as defined by the World Health Organization, include service delivery; health workforce; health information; medical products, vaccines and technologies; financing; and leadership and governance [20]. In addition to the tangible elements of health systems, Sheikh et al. [21] argue that ‘software’, such as values, norms, and power, that “*guide actions and underpin the relationships amongst system actors and elements are also critical to overall health system performance*”.

The publication of this special issue has two main aims: first, to highlight where our approaches have been able to contribute to policy-uptake in Pakistan, and second, to further contribute to the dissemination of evidence from Pakistan, stimulating MNH policy and practice reforms. The nine research papers and the third commentary included in this issue stem from projects funded by the Maternal and Newborn Health Programme Research and Advocacy Fund (RAF), a programme implemented within the health system context in Pakistan and which aimed to espouse the principles of evidence, advocacy, and dissemination described above.

RAF was set up as a grant fund in 2008 to work with researchers and civil society organisations to influence pro-poor policy and practice reform on MNH in Pakistan. In addition to providing grants, RAF provided capacity building support to researchers and civil society organisations, for example, technical advice during the proposal development phase on research methods, advocacy strategies, and gender and inclusion, and communication approaches. The programme also held consultations and reviewed gaps in evidence to identify topics for advocacy. Overall, 56 research and advocacy projects were funded, which provided a range of evidence on critical MNH issues, influencing over 30 different policy and practice issues. In addition to policy reforms documented in the special issue, we witnessed RAF-funded projects contributing to changes in health worker curriculum, legislation on breastfeeding and nutrition being passed in Sindh, Punjab, Balochistan, Khyber Pakhtunkhwa, and Azad Jammu and Kashmir, and increased government commitments to family planning targets in line with Family Planning 2020.

## Profiling advances in research, advocacy, and uptake of evidence in Pakistan

In the articles included in the special issue, critical health system issues compromising MNH outcomes in Pakistan are discussed, for example, service delivery components, demand creation, equitable access, transportation interventions for improved referrals, availability of medicines and equipment, and health workforce needs. In addition to these tangible elements, the ‘software’ of the health system, i.e. the power and the political and social contexts, is also represented in the collection.

The first two commentaries from Ghaffar et al. [22] and Bhutta et al. [23] provide an overview of Pakistan in terms of its contributions to global maternal and child health policies and strategic options. Sarwar et al. [24] discuss RAF-supported advocacy efforts relating to misoprostol and chlorhexidine use. Despite the available evidence base on the use of misoprostol for treatment and prevention of postpartum haemorrhage and chlorhexidine for the prevention of newborn sepsis, the availability and utilisation of these medicines was limited in Pakistan. A group from Mercy Corps identified the knowledge and practice gap by evidence review and field consultation, before facilitating a multi-stakeholder, consultative process involving policymakers, policy implementers, and opinion leaders. Focused advocacy efforts resulted in a number of policy gains such as the inclusion of the medicines in the essential drug list throughout the country. The authors call for continued focus on the scale-up of appropriate use of chlorhexidine and misoprostol and the use of multi-stakeholder forums for future policy initiatives.

Pakistan’s Community Midwives (CMW) programme has introduced a new cadre of skilled birth attendants in the community. Similar to Indonesia’s Village Midwives, who had the right to establish private practices, Pakistan’s CMWs were expected to establish private practices after 2 years of government financial support. Many Indonesian village midwives abandoned public service in favour of the more lucrative private practice and the maternal mortality ratio stagnated; three papers examine this high profile initiative. Mumtaz et al. [25] used a population-based survey in two districts in Punjab province to examine women’s uptake of CMWs. They showed that individual characteristics, such as education or socio-economic status, have no direct bearing on use of a CMW in childbirth. What emerged as an important factor was the context in which a woman lived, suggesting that social norms and standards of development may need to be challenged and changed to enhance demand for skilled birth attendants. Zafar et al. [26] assessed the relevance of the current CMW programme policy and explored community members’ preferences regarding birthing place. Based on community members’ responses to a hypothetical question about preferred birthing place, the authors argue that community members prefer a birthing station, which is accessible within their community and adequately equipped for childbirth. After results of this study were disseminated within Pakistan, the National Maternal, Newborn and Child Health (MNCH) Programme introduced birthing stations, changing the way the MNCH Programme is being delivered. A new initiative of training CMWs on family planning service provision in rural settings is discussed by Hameed et al. [27], by comparing the CMW delivery model to one involving a network of private providers who became franchises of a local brand, supported by training and advertising. ‘Social franchising’ similar to ‘business franchising’ is a method of expanding service delivery points for social goals by influencing private sector providers [28]. Using a quasi-experimental study design, the study demonstrated that the two delivery models were able to achieve high continuation rates of intra-uterine contraceptive devices, a long-acting reversible contraceptive that is underutilised in Pakistan.

Contracting out is another method of quickly expanding service availability. Zaidi et al. [29] used a mixed methods approach to present comparative advantages of contracting out in provision of MNH care in Pakistan. Drawing on a household survey and facility assessment data, the authors showed higher utilisation of MNH care services in the catchment population of rural health units contracted to an NGO; however, the results suggest that the users were largely the educated and the wealthy. The authors argue that targeted measures to promote behavioural changes and to increase demand are needed to have a meaningful impact on utilisation of care in remote settings.

Inequity in access and the barriers to Emergency Obstetric and Newborn Care services in Sindh province are discussed by Ansari et al. [30]. In addition to financial and geographical barriers, the authors highlight unfavourable attitudes and practices of healthcare providers as important barriers to seeking care from public sector hospitals. Further, they suggest that caste-, religion-, and ethnicity-based discrimination operates at public sector hospitals, leading women to seek care elsewhere. Discrimination of certain groups of women at public sector hospitals, compounded by the dual practice of healthcare providers in the public and private sectors, appears to work synergistically, diverting women to seek care from private sector practitioners.

The issue of caste-based discrimination and social exclusion is further discussed by Aziz et al. [31], who critically examined the influence of the political and social contexts on community participation in Punjab. Using various participatory rural appraisal tools, the authors identified and examined social hierarchies and formal and informal ‘spaces’ where health activities would take place in rural communities. Their analysis showed that programme planners’ predilection for notable community leaders in gaining support for community-based health programmes often results in the exclusion of vulnerable groups from health education. Caste-based boundaries were less rigid amongst men. Health-related information trickled down from higher to lower caste men, while women of lower caste, impinged by the rigid caste-based boundaries, experience barriers to informal information exchange with women of the better-off castes. As the structures and designs of MNH programmes reinforced the existing power dynamics, the authors argue that the systematic factors need to be carefully considered in designing MNCH programmes and call for the redefining of programme structures and strategies in favour of vulnerable population groups.

Pivotal to the functioning of a good maternity healthcare system is a referral system. Mian et al. [32] reviewed various transportation initiatives in Pakistan, which aimed to improve patient referrals. They assessed the potential of scaling up the interventions for improved patient referrals. The appraisal to assess scalability was based on seven criteria, including credibility of the intervention, relevance to population demand, and easy transferability. Amongst four categories of initiatives (i.e. community-based, facility-based, public sector, and voucher schemes), the authors found that community-based interventions had the potential for scale-up because of the simple design and community ownership and participation, which will ensure sustainability.

Human resources, another important component of a health system, are examined by Mir et al. [33] for this special issue. High attrition of healthcare providers in rural areas is a critical issue facing the health system in Pakistan. Drawing on the national survey of public-sector healthcare providers, the authors identified the factors associated with the healthcare providers’ willingness to leave government services. The study found that healthcare providers who are dissatisfied with performance review process or salary and working in certain areas of the country (such as Balochistan and Azad Jammu and Kashmir), appear to be more willing to leave. The authors recommend the development of an incentivising mechanism to accommodate healthcare providers in harsh working conditions as well as administrative reforms to revise the performance evaluation system incorporating new job-based indicators.

We have included a short article by Majrooh et al. [34] to demonstrate results of a facility assessment in Punjab province. The study showed clear gaps in service provision for antenatal care. The results have had a significant impact in Punjab province as the government revised the kits for CMWs based on findings from the assessment. Data from a simple cross-sectional facility assessment may be useful in demanding resources for infrastructure and equipment needed for quality healthcare services.

## Looking forward

We began this article by considering the essential and interlacing influences of research, dissemination, and advocacy for policy uptake. Understanding of how these groups of activities and actors can and should work together has grown considerably in the last decade [35, 36]. Simply conveyed by its name, the RAF was established as an original and unique initiative in its time, consequently playing an important role in bringing research and advocacy together and helping to bridge the gap between evidence and decision-making by putting dissemination, communication, and advocacy at the core of all of its activities.

The articles in this supplement highlight three considerations for the future: the growing importance of implementation research, the crucial need for participation and ownership, and the recognition that policymaking can be ‘informed’ by rather than ‘based-on’ evidence.

## Implementation research

We are in a very different place in MNH today compared to where we were two decades ago. The last 20 years has seen a burgeoning of knowledge generation in ‘what works’ in MNH, including clinical studies on technological innovations and randomised controlled trials on complex interventions [37-40]. Despite documented progress in reducing maternal and child mortality, however, our failure to make sufficient improvement is related to the lack of knowledge in understanding ‘why’ and ‘how to’. This is where implementation research comes to the forefront. Implementation research has been acclaimed as a “*powerful tool for capturing and analysing information in real time, allowing for the assessment of performance, for example, and facilitating health systems strengthening*” ([41], p. 8). It aims to capture information to explain what is happening and why [42], especially for finding solutions to complex problems in the ‘real world’ of programme implementation and policymaking [43]. Qualitative and mixed methods studies play an important role in ensuring that the realities of people’s experiences are captured. In prioritising the funding of implementation research (and in particular, not only funding primary research using randomised control trials, but focusing on mixed methods and qualitative research studies [44]), RAF has made an important start to developing a ‘research culture’ in Pakistan. It is important to maintain this momentum for the future.

Implementation research is not free from limitations and challenges, however. It will certainly present the same problems as other types of research, and most obviously, findings of implementation research need to be taken up by the implementers to close the gap between evidence generated by the implementation researchers and practices. Efforts are still needed to improve methods of dissemination. Researchers and implementers must come together to strategize to achieve the desired research objectives. RAF’s experience suggests that engaging stakeholders at the early stages of research development for the identification of priority topics and preferred research designs is important for policy uptake and increased ownership (which is discussed further below). In terms of methodologies, implementation research is a relatively new field. There is no consensus on optimal methodology for implementation research and interdisciplinary/multidisciplinary approaches are needed, drawing on quantitative, qualitative, and mixed methods. Discipline-specific questions (e.g. [45]) are also emerging. New methods are developed/borrowed from other disciplines [43]. Training of a new generation of researchers is needed to tackle research questions arising in this new field.

## Participation and ownership

In resource-constrained settings, there is a special need to prioritise initiatives for research and advocacy on contextually-relevant priorities. To do so, wide and inclusive participation of stakeholders is necessary [46, 47]. Participation can create openness, transparency, and accountability, creating a ‘pull’ for mutual learning and discussion [48]. This opens out the possibility of developing policy and having shared ownership of decisions made. In addition, participation allows identification of local priorities based on expert, local knowledge. By empowering national stakeholders to have greater ownership of the priorities for research and advocacy, the chances of improving uptake of findings into decision-making processes for future policy and programming are increased. The principles of participation are reflected in the way RAF played a role in opening dialogue between policymakers, researchers, and advocates. As shown in this special issue, a number of tangible changes to health policy and practices have been made following dissemination of results from RAF-funded research and advocacy projects. RAF acted as a catalyst in its short life in Pakistan.

We also acknowledge the limitations and challenges associated with stakeholder participation and ownership. When engaging with stakeholders, not everyone’s view can be represented, as ‘stakeholders’ are non-homogenous groups of people with differing motives and purposes. Participation is a highly political process where existing power relations may be reinforced [31]. Though often overlooked, it is important to engage stakeholders at a local and community level, particularly to feedback research findings to participants and communities. RAF was able to play a part in facilitating participation to some extent: it acted as a bridging organisation creating forums for dialogue or partnering researchers with civil society (such as NGOs). By supporting qualitative and mixed methods studies that capture the voices of the poor, RAF supported the ‘participation’ of ordinary citizens [44].

## Evidence-informed policymaking

Our paper began with the idea of evidence-based policymaking. However, it is well recognised that policy is influenced by more than just evidence, with politics, lobby groups, media, and chance playing a part [49,50]. The types of evidence that are considered in policymaking are varied. Multiple sources of knowledge and information and various types of research-based evidence interact with the belief and value systems of policymakers and influence their decisions, sometimes in subtle and indirect ways [51]. Organizational values and cultures and decision-making processes vary from one setting to another; hence, contexts inevitably play a significant role in how decisions are made. Making policy decisions solely based on evidence may simply be an aspirational goal. Understanding how evidence can inform policymaking will help researchers identify types of research evidence useful for policymakers and work together to improve public health policies and eventually health outcomes. This means that what has been done by RAF is only a start and questions of how to build on its legacy need to be considered before the gained momentum is lost.

## The future

There will be no quick fixes in tackling such complex issues in the future of Pakistan. The building of robust healthcare systems is an area where many low- and middle-income countries have been underperforming [52]. These systems will require stronger health infrastructure, more health workers, greater access to medical products, more responsive health services, and more equitable access to these services. It has been estimated that the cost for health system strengthening will be about $30bn annually for the next few decades. The upside is that such funds can be sourced from a combination of aid and domestic spending given the economic growth and productivity of many low- and middle-income countries [53], provided they increase their domestic health spending. Impressive gains have been made in moving towards reaching MDG-4 and −5. RAF, as well as the many groups, academia, NGOs, and government programmes not directly funded by RAF but which dared to step outside their comfort zone and embrace participation in RAF’s aims, have made key contributions, as attested by this collection of papers. The challenge for the future will be to build on these foundations to welcome a new era of health, wealth, and growth for Pakistan.

## Abbreviations

CMW: Community midwives
MDGs: Millennium Development Goals
MNCH: Maternal, newborn and child health
MNH: Maternal and newborn health
NGO: Non-governmental organisations
RAF: Research and Advocacy Fund
